# Childhood Lead Poisoning in China: Challenges and Opportunities

**DOI:** 10.1289/ehp.1307558

**Published:** 2013-10-01

**Authors:** Chong-huai Yan, Jian Xu, Xiao-ming Shen

**Affiliations:** 1 Deputy Director of MOE–Shanghai Key Laboratory of Children’s Environmental Health, Xinhua Hospital, Shanghai Jiao Tong University School of Medicine, Shanghai, China; 2 MOE–Shanghai Key Laboratory of Children’s Environmental Health, Xinhua Hospital, Shanghai Jiao Tong University School of Medicine, Shanghai, China

It is well known that children < 7 years of age are uniquely susceptible to lead poisoning because of their constant hand-to-mouth behaviors, their immature central nervous systems, and their rapidly developing bodies (Gavaghan 2002; Lanphear et al. 2005; Shen 1996; Tong et al. 1996, 1998). Although exposure to lead has decreased in many countries, significant concerns remain about continued exposure of children to lead. From the end of the 1990s through 2009, China’s average blood lead levels in urban children 0–6 years of age decreased from 7–10 µg/dL to 2.5–6 µg/dL (Peng et al. 2011; Qi et al. 2002). The prevalence of children with higher blood lead levels (≥ 10 µg/dL) decreased from 30–50% to 1.5–15% (Luo et al. 2011; Xiong et al. 2011). The decline in blood lead levels appears to be associated with national efforts to decrease lead pollution, including the phaseout of leaded gasoline; a transition from coal fuel to diesel, natural gas, and other clean energy alternatives; and closing or merging heavily polluted enterprises. In addition, many lead-polluting industries have migrated from large cities to middle-size and small cities and to rural areas, often from eastern China to western China.

However, there has also been a rapid development of automobile and information industries and an increased demand for lead-acid batteries in China during the last decade. China has also experienced a significant expansion in galena mining, lead smelting, battery production and recycling, e-waste disassembly and recycling, metal processing, production of lead-containing chemicals, cable manufacturing, and production of wire rope. In addition, there has been a substantial increase in the number of small family businesses that use lead-containing products. Although an overall decline in children’s blood lead levels has been observed over in years, children’s exposure to lead is still common in many Chinese cities.

Industrial pollution is clearly one of the most important causes of lead poisoning among children in China. However, other significant sources of exposure may cause lead poisoning in children. For example, young children may ingest or swallow toys or other items or prescribed medicines containing lead. Some lead compounds, including lead tetraoxide (red lead), lead monoxide (yellow lead), and basic lead carbonate are used in folk remedies for convulsions and carbuncles and as astringents. Many cases of clinical lead poisoning are caused by topical or oral administration of lead-containing compounds in the treatment of vitiligo, eczema, epilepsy, diarrhea, cough, asthma, oral diseases, and intestinal parasites. In some areas of China, newborns or infants are still treated with red or yellow lead powder for skin care, either with lead powder alone or powder mixed with commercially available talcum powder. Lead poisoning in children can also be caused by using lead powder to treat mouth ulcers. Sometimes cooking wine or water stored in lead-containing pots is used to prepare food or reconstitute milk power, which can result in significant exposure to lead.

At the present time, blood lead screening is the only effective way to identify lead-poisoned children. Every year, tens of thousands children are screened in China, and a considerable number of children with elevated blood lead and lead poisoning are identified. However, screening for blood lead level occurs typically in response to requests from parents and not as part of an overall examination. Thus, a large number of children may have lead poisoning that is undetected, and therefore they do not receive timely diagnosis and treatment. Unfortunately, pediatricians can misdiagnose or overlook cases of lead poisoning because they lack training in the prevention and treatment of childhood lead poisoning.

At the present time, a number of policies and measures could be implemented to promote the prevention and control of childhood lead poisoning in China. Regulatory policies need to be put in place to reduce lead emissions from numerous lead-related industries. There is a need to develop new and renewable energy sources, including wind, solar, water, and nuclear power; reduce coal consumption; and attenuate air pollution from the thermo-power–generation process. There is a need to improve quality control systems for blood lead screening and blood lead testing; promote nationwide implementation of unified blood lead testing techniques and methods; and increase the overall implementation of blood lead testing in primary health care settings. Every child ≤ 6 years of age should have the opportunity to receive blood lead testing. For children living in lead-contaminated areas, special screening programs should also be developed. Finally, the public and pediatricians in China need to be educated about the prevention and treatment of childhood lead poisoning.

The experience of China, the United States, and other countries supports the idea that childhood lead poisoning is preventable. Although significant improvement has occurred in China over the last 20 years, many challenges remain. Coordinated and sustained efforts will be required to lessen the impact of exposure to lead on Chinese children now and in the future.
